# Climate change in fish: effects of respiratory constraints on optimal life history and behaviour

**DOI:** 10.1098/rsbl.2014.1032

**Published:** 2015-02

**Authors:** Rebecca E. Holt, Christian Jørgensen

**Affiliations:** 1 Department of Biology, University of Bergen, PO Box 7803, Bergen 5020, Norway; 2 Uni Research, PO Box 7810, Bergen 5020, Norway

**Keywords:** oxygen, aerobic scope, temperature, fitness

## Abstract

The difference between maximum metabolic rate and standard metabolic rate is referred to as aerobic scope, and because it constrains performance it is suggested to constitute a key limiting process prescribing how fish may cope with or adapt to climate warming. We use an evolutionary bioenergetics model for Atlantic cod (*Gadus morhua*) to predict optimal life histories and behaviours at different temperatures. The model assumes common trade-offs and predicts that optimal temperatures for growth and fitness lie below that for aerobic scope; aerobic scope is thus a poor predictor of fitness at high temperatures. Initially, warming expands aerobic scope, allowing for faster growth and increased reproduction. Beyond the optimal temperature for fitness, increased metabolic requirements intensify foraging and reduce survival; oxygen budgeting conflicts thus constrain successful completion of the life cycle. The model illustrates how physiological adaptations are part of a suite of traits that have coevolved.

## 1. Introduction

Owing to climate change, water temperatures have increased in marine and freshwater habitats around the world. Current projections predict a mean rise in temperature of 2–4°C globally by the end of this century, although locally the increase can be higher [1]. For aquatic ectotherms, this may pose challenges of sufficient oxygen uptake to sustain their metabolic demand. Compared with air, water contains 23 000 times less oxygen per mass [2], which necessitates energetically expensive ventilation. The high thermal conductivity of water makes it challenging for aquatic ectotherms to maintain an internal temperature that differs from the surrounding water [3]. As temperatures increase, the oxygen content of water drops while metabolic costs rise. This dual challenge is particularly problematic as almost all of the heat associated with climate warming is taken-up and stored within the world's oceans [1].

The theory of oxygen- and capacity-limited thermal tolerance (OCLTT) describes how maximum oxygen uptake constrains overall metabolism for aquatic organisms, and how this constraint varies in response to stressors such as temperature and hypoxia ([4], see [5] for terrestrial parallel). In particular, aerobic scope is defined as the difference between maximum oxygen uptake and standard (resting) metabolic rate [6], and all activities that the organism performs to achieve fitness must fit within this aerobic budget [7].

The OCLTT simply assumes that the temperature at which aerobic scope is maximized is the temperature that will also maximize fitness. This has been challenged by measurements showing that aerobic scope may have no optimum but increase up to lethal temperatures [8]. This suggests that aerobic scope is not the central constraint on performance [9], and that additional factors influence fitness.

If locomotion, digestion and growth were all running at their maximum rate, then the requirement for oxygen would greatly exceed aerobic scope [7]. With a finite oxygen budget, some prioritization must take place [10], which results in trade-offs mediated by oxygen availability. Thus, temperature-dependent physiology scales the overall oxygen budget through its effects on aerobic scope. Within that scope, fitness-related processes are subject to further trade-offs that may depend on temperature and ecology. Using state-dependent bioenergetics, we explicitly model the missing link between respiratory physiology and fitness. We show that the optimal temperature for fitness is lower than that of aerobic scope, and emphasize how limited oxygen budgets cause trade-offs and act as both constraint and driver of change.

## 2. Material and methods

We use a state-dependent energy allocation model that predicts optimal behaviour and life-history strategies in response to environmental temperature [11], with parameters describing the Northeast Arctic (NEA) stock of Atlantic cod (electronic supplementary material, figure S1) [11]. We focus on adult cod, modelling age 1 year onwards. The model is based upon OCLTT with focus on temperature-dependent bioenergetics and aerobic scope as the central constraints. We include a number of trade-offs: survival is reduced by intense foraging ([11–13]; see electronic supplementary material), high oxygen consumption and high reproductive investment. In particular, the lack of free aerobic scope increases mortality as fish are less able to escape predators [13]. Furthermore, predation declines with body size, which links long-term consequences of energetics and growth to life history and fitness [11]. The only temperature-dependent functions in the model are maximum oxygen uptake and standard metabolic rate (electronic supplementary material, figure S2).

Optimal life-history strategies are found by optimization using dynamic programming [14] and comprise values for foraging behaviour and energy allocation that maximize the expected lifetime reproductive output. Optimal strategy values are found for each combination of the individual states (age and length) and the current food availability in a stochastically variable environment. The optimal strategy is then simulated in a population, through which emergent properties such as growth, maturation, reproduction and survival are recorded. We step-wise re-run the model at increasing temperatures to find new optimal life-history strategies in a new and warmer environment. For a full model description, see [11]. In this paper, we run simulations over a wide range of temperatures (2–20°C) to assess the broad-scale relationship between temperature and fitness.

## 3. Results

**Figure 1.**
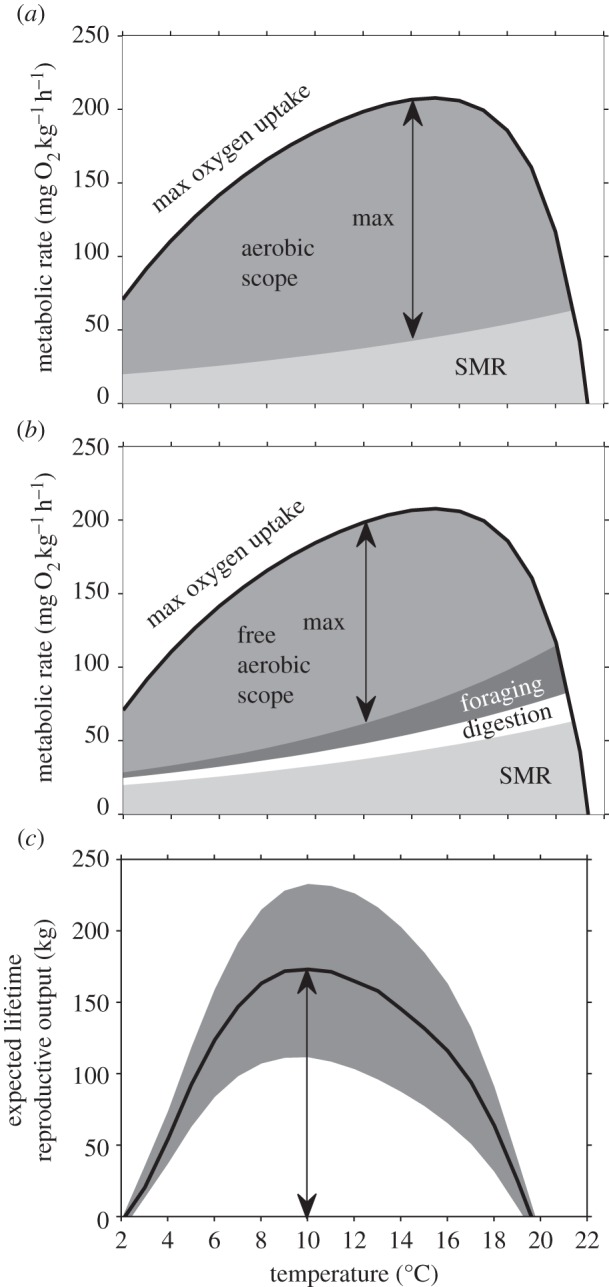
Predicted effects of respiratory constraints and survival trade-offs on cod fitness under climate warming. In the definition of aerobic scope (*a*) processes such as foraging and digestion required for long-term survival are not included ((*b*) see electronic supplementary material). These overhead costs increase with temperature, causing fitness (*c*) to decline more rapidly at warmer temperatures than predicted by aerobic scope alone. In (*c*), the central line denotes the population mean and shaded grey areas within-population variance due to environmental stochasticity.

In the model, the temperature that maximizes aerobic scope is 14°C for NEA-cod (figure 1*a*). This, however, only applies to the short-term equilibrium where standard metabolic rate is the only cost. If individuals are to maintain energy balance in the longer term they also need to forage and digest, which incurs additional oxygen demand and can be thought of as overhead costs. By incorporating these costs, the temperature that maximizes free aerobic scope available for growth and reproduction is reduced to 12°C (figure 1*b*).

Natural selection will favour those strategies that most efficiently convert free aerobic scope into reproductive output. By encompassing this life-history consideration, the optimal temperature for fitness for NEA-cod is 10°C (figure 1*c*). This is 4°C lower than the optimal temperature suggested by OCLTT based on the temperature at which aerobic scope peaks.

**Figure 2.**
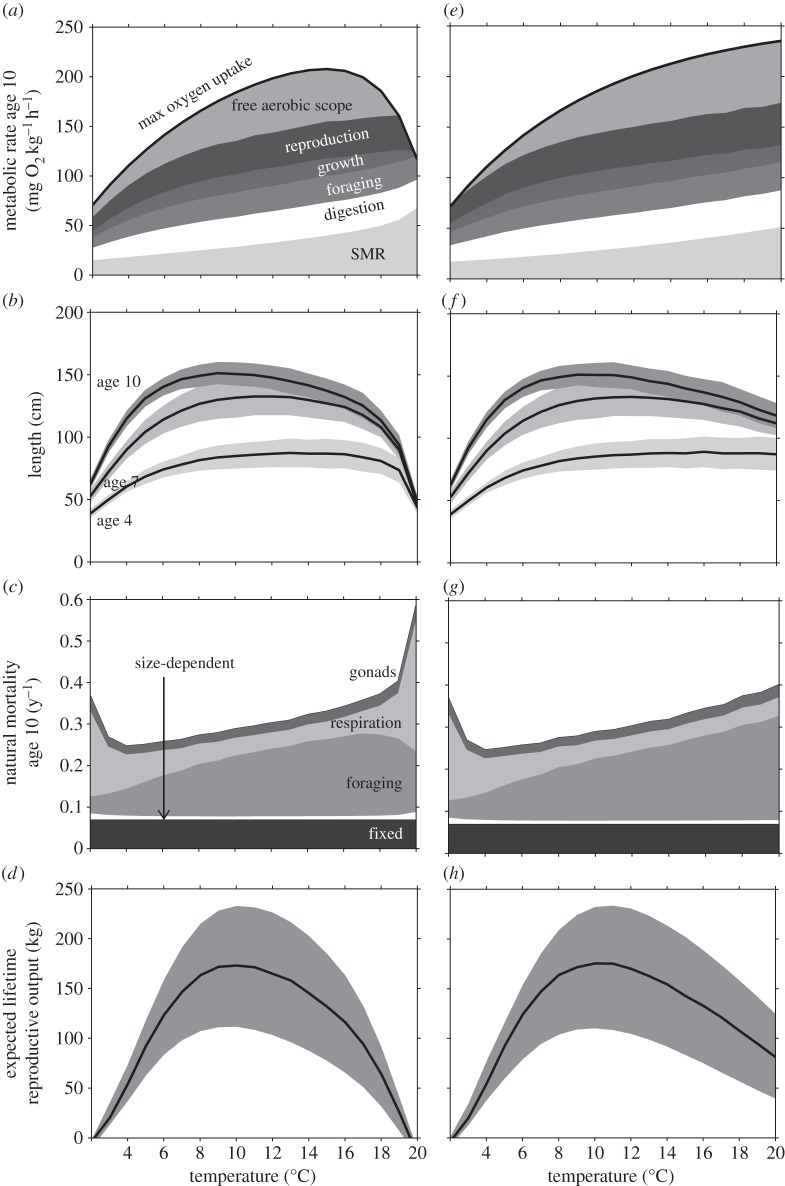
Consequences of optimal temperature adaptations for (*a*) oxygen use (see electronic supplementary material), (*b*) length, (*c*) natural mortality, and (*d*) expected lifetime reproductive output shown for age-10 cod under climate warming. Panels *e*–*h* are the same as *a*–*d* but use an ever-increasing function of maximum oxygen uptake (electronic supplementary material figure S2). For length and fitness, mean and variance are shown as described in legend to figure 1.

When considering all activities that the organism performs over the full temperature range, NEA-cod has a complicated pattern of aerobic scope budgeting that changes with temperature (figure 2*a*; see electronic supplementary material). With higher temperatures, overall metabolism goes up, which drives an increased demand for foraging (electronic supplementary material, figure S3*c*), which increases the associated predation risk (figure 2*c*). At very high temperatures, 16–20°C, foraging mortality declines and respiration mortality is predicted to increase (figure 2*c*), indicating that lack of aerobic scope has taken over as the dominant constraint. Aerobic scope is most fully utilized at low and high temperatures (figure 2*a*), thus lowering the ability to escape predation, causing increased mortality (figure 2*c*). At these temperature extremes, lack of aerobic scope puts constraints on growth (figure 2*b*), reproduction and survival (figure 2*d*). At intermediate temperatures, aerobic scope seems to have less influence and trade-offs related to foraging appear to be shaping optimal life histories.

Recent studies show that in some species maximum oxygen uptake curves lack a temperature optimum but increase up to lethal temperatures [8,9], calling the role of respiratory constraints into question. We therefore performed sensitivity analyses using an ever-increasing maximum oxygen uptake function (figure 2*e–h*; electronic supplementary material figures S2 and S3*g–l*). Despite the increased amount of aerobic scope available (figure 2*e*), the optimal temperature for fitness remained at 10°C (figure 2*h*), much driven by natural mortality responding similarly as in the model runs, with a peaking aerobic scope curve. This suggests that aerobic scope does not predict performance at high temperatures, and that behaviour, life-history traits and their trade-offs must be considered to make predictions about temperature-dependent fitness.

## 4. Discussion

We study the effects of climate warming on NEA-cod optimal life history and behaviour, focusing on temperature-dependent effects on aerobic scope as a key constraint and as identified by OCLTT [4]. However, OCLTT assumes that the temperature that maximizes aerobic scope is also the temperature that maximizes performance and fitness, whereas our model predicts optimal temperatures for growth and fitness that lie well below that for aerobic scope.

The basic cost of existence—standard metabolic rate—typically increases with temperature, which puts added requirements on foraging and digestion. This increases mortality incurred through foraging, under which natural selection would favour accelerated life histories with faster growth, earlier maturation and higher reproductive investment. This is achieved through even more intense foraging, which further accelerates the process and causes a fitness optimum at colder temperatures.

Based on their thermal distribution, it has been suggested that adult Atlantic cod cannot live in regions where the average annual temperature exceeds 12°C [15]. Fitness, however, is also predicted to be high at 14–16°C, much higher than at temperatures of 2–4°C where cod is abundant, probably because it is one of few species that are successful cold-water predators. The absence of cod in warmer waters may be a consequence of ecological factors that we do not include within the model: competition from warmer water specialists, temperature effects on food availability, better escape behaviour of warm-water prey or effects on early life stages of cod. Additionally, species may behaviourally avoid environments near their upper thermal limit as a safety margin [16,17].

Our predictions for lifetime gonad production are consistent with the population-level observation of recruitment increasing by 40–50% per 1°C increase of sea surface temperature for NEA-cod [11,18,19], suggesting that model predictions resemble population-level responses to climate warming even when we do not consider early life stages.

Using an ever-increasing curve for maximum oxygen uptake leaves predictions for fitness virtually unchanged up to 14°C. The concern that aerobic scope is a poor predictor of performance at high temperatures has been raised for other species, including barramundi (*Lates calcarifer*) [8], freshwater shrimp (*Macrobrachium rosenbergii*) [20] and Atlantic halibut (*Hippoglossus hippoglossus*) [21], where aerobic scope was available up to their critical temperatures, at which point death ensued. We perhaps need therefore to step away from aerobic scope as a unifying principle and move towards the concept of multiple performances and multiple optima as a way to integrate effects of physiological functions with different temperature sensitivities [9,21] through to their effects on behaviour, life histories and fitness.

This mechanistic modelling framework can be adapted to other marine teleosts, particularly other boreal and temperate species. As we include an explicit respiratory physiology component, it allows flexibility to include additional drivers, such as the effects of ocean acidification, hypoxia or pollution on increased respiratory costs [4,22], as well as effects of fishing mortality. The integrating effects of multiple drivers on respiratory physiology are already prominent within current literature [22]; our mechanistic model could take this further to assess effects on life-history traits and fitness.

Our predictions highlight the need to use caution when inferring optimum temperatures for performance and fitness derived from metabolic data alone. There is no doubt regarding the importance of aerobic metabolism in daily activities and for fitness. However, organisms are complex; they possess a suite of traits, not only physiological but also behavioural. These have co-evolved and may continue to do so, and thus must be viewed as a whole when predicting the effects of climate change on fish.

## Supplementary Material

Supporting Information: Climate change in fish: effects of respiratory constraints on optimal life-history and behaviour

## Supplementary Material

Matlab Model Code, Climate change in Fish: Effects of respirometry constraints on optimal life-history and behaviour

## Data accessibility

Matlab code is available in electronic supplementary material.
