# Supplementary material for: Climate change in fish: effects of respiratory constraints on optimal life history and behaviour
Source: Biol Lett. 2015 Feb;11(2):20141032. doi: 10.1098/rsbl.2014.1032 (PMC4360111; doi:10.1098/rsbl.2014.1032)
Supplement: Supporting Information: Climate change in fish: effects of respiratory constraints on optimal life-history and behaviour [file rsbl20141032supp1.pdf]

Electronic Supplementary Material

Climate change in fish: effects of respiratory constraints on optimal life-history and behaviour

Rebecca E. Holt and Christian Jørgensen

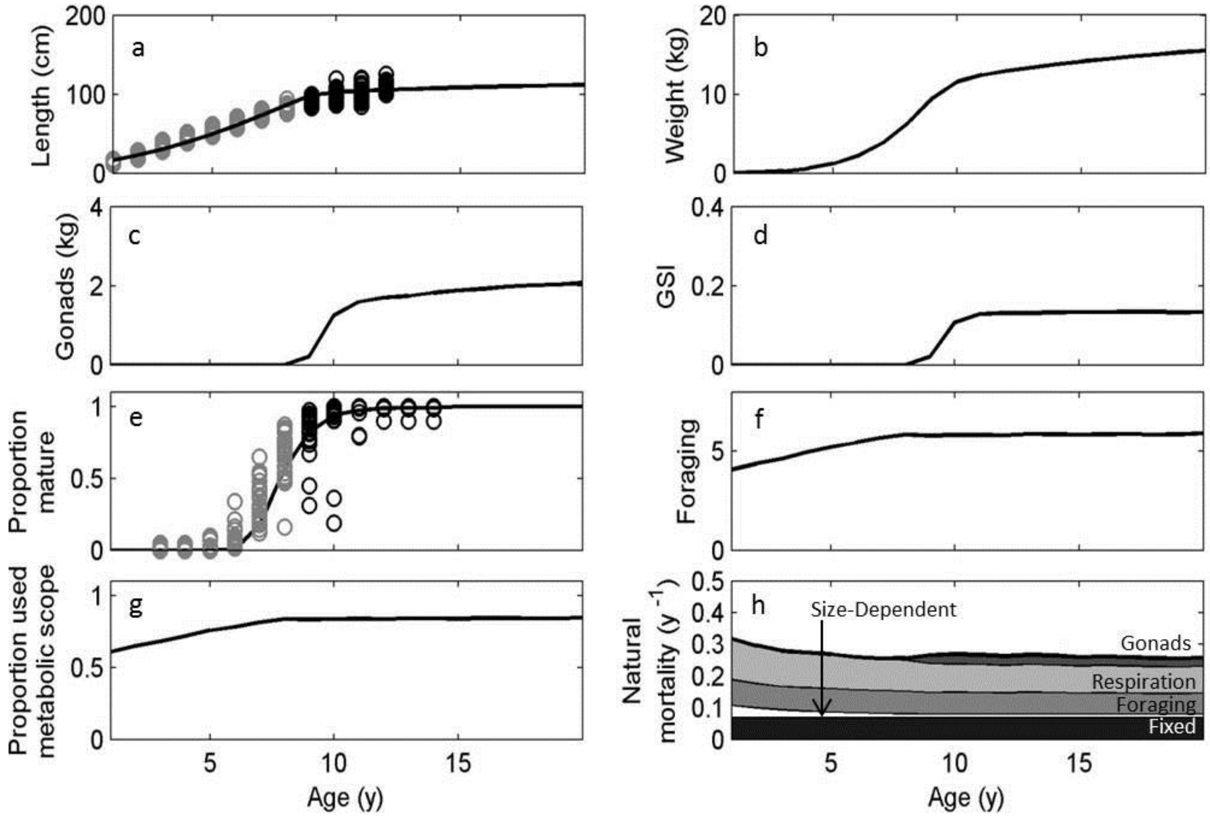

Figure S1. Optimal life history at 4°C for the Northeast Arctic stock of Atlantic cod. Predicted mean lifetime trajectories of body length (a) weight (b), gonad weight (c), gonado-somatic index (d), proportion mature (e), foraging behaviour (f), proportion of used metabolic scope* (g), and annual rates of natural mortality (h). Predicted length-at-age (a) and proportion-mature-at-age (e) are compared with the International Council for the Exploration of the Sea (ICES) survey data from the Barents Sea (grey open circles) and Lofoten (black open circles; ICES, 2012). *All metabolic processes are summed and divided by maximum oxygen uptake to find the degree to which metabolic scope is used (Please see Holt & Jørgensen, 2014 for details).

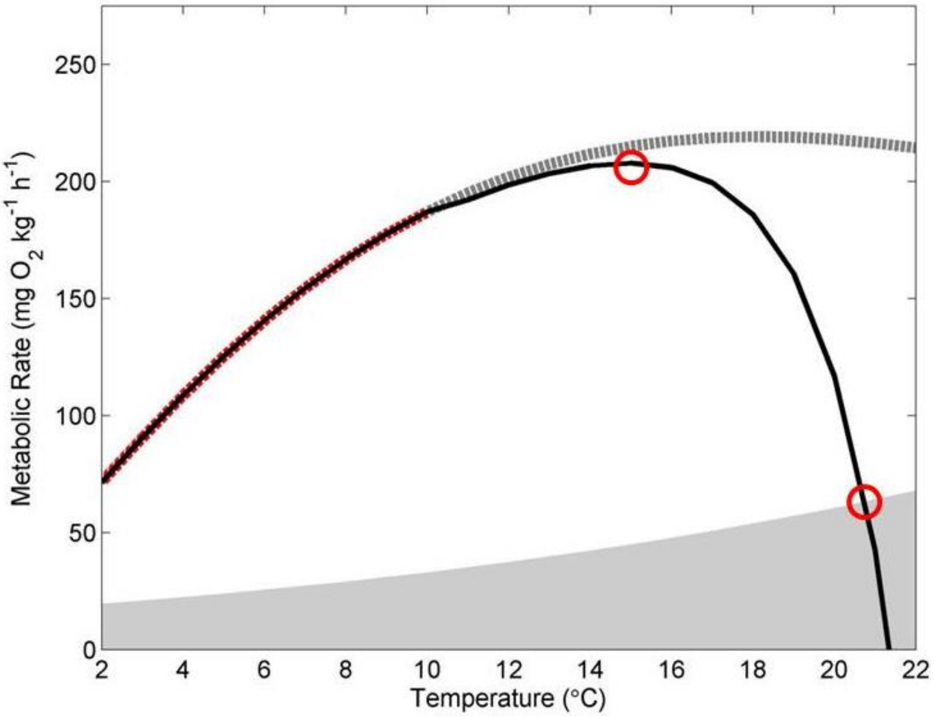

Figure S2. Parameterisation of maximum oxygen uptake curve Black solid line indicates the modified *V*max relationship used in this study. We parameterised it as follows. *V*max (Maximal aerobic metabolism) was experimentally determined by Claireaux *et al*. (2000) for the temperature range indicated by the red dashed line. We assumed a peak in oxygen uptake coinciding with the peak temperature for growth at 15°C for Atlantic cod (Jobling, 1988) (red circle). We further assumed that oxygen uptake is identical to standard metabolic rate (B_{SMR}, grey shaded area) (Clarke and Johnston, 1999) at the critical temperature for Atlantic cod (21°C, Jobling, 1988), indicated by second red ring. Grey dashed line is Vmax extrapolated from Claireaux et al. (2000) beyond the temperatures used in their experiments, and was used for sensitivity analysis (Fig. 2d-f).

Explanation of method used to calculate Fig. 1b (main text)

Overhead costs in Fig. 1b are calculated (for a 3kg fish) as the foraging and digestion needed to sustain the increasing costs of SMR at higher temperatures to maintain energy balance using bisection iteration.

Explanation of method used to calculate Fig. 2a,e (main text)

For details and parameter values please see Holt & Jørgensen (2014), symbols used below refer to their use in that paper. In the model, we quantify the following bioenergetics components for age 10 fish: standard metabolic rate (B_{SMR}, of somatic mass), specific dynamic action (B_{SDA}), cost of foraging activity (B_{φ}), the energetic costs of swimming), somatic growth (cost of tissue production and metabolic work associated with energy conversion), reproductive costs (maintenance metabolism of gonads, tissue production and metabolic work associated with energy conversions). The model calculates growth costs together, we split them between somatic and gonadic growth to reveal life-history dynamics. As fish differ in size at age 10, the values are plotted as coefficients (per kg body mass) and compared to the maximum oxygen uptake curve used in our present study (Fig. S2).

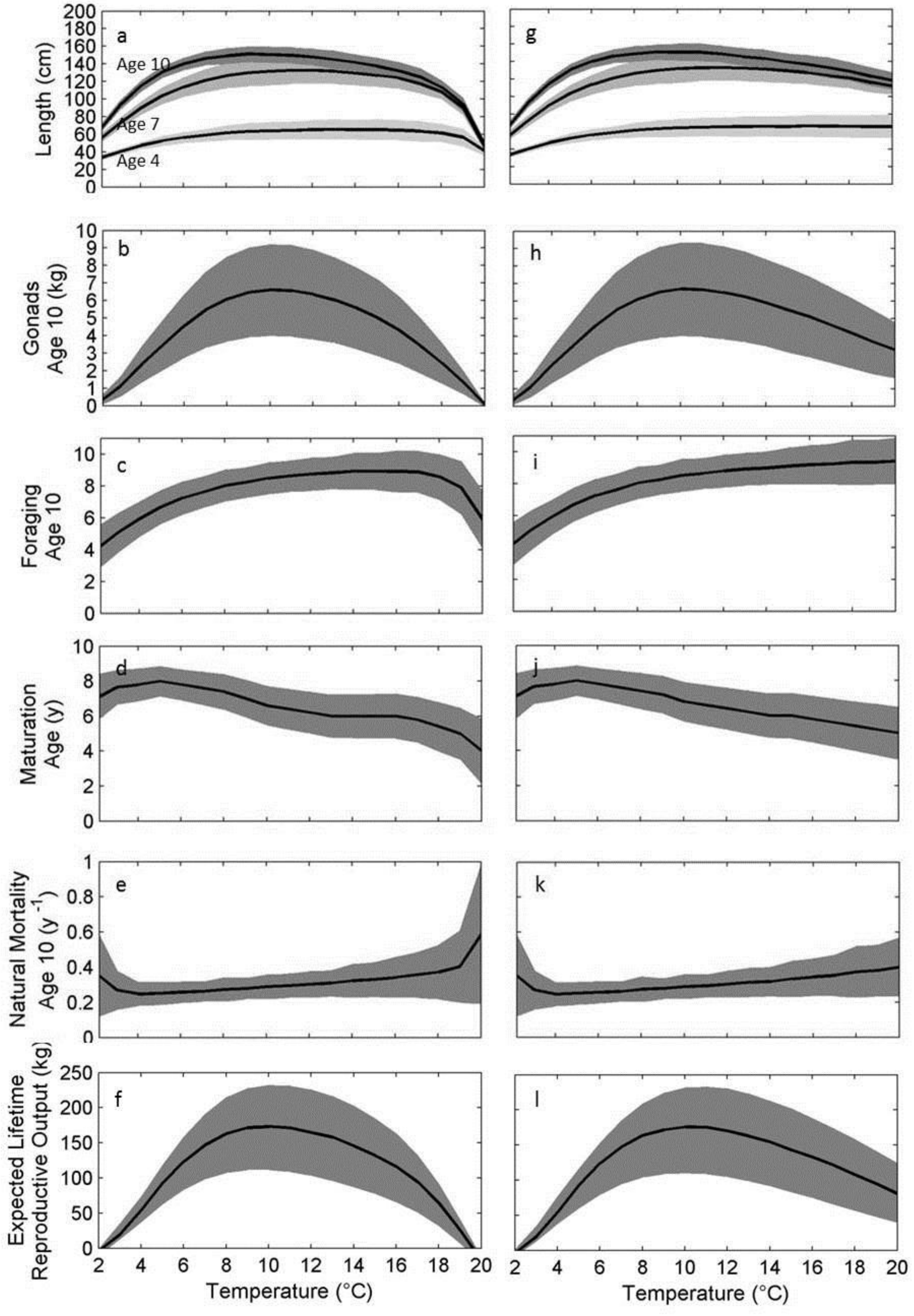

Fig S3. Predicted lifetime trajectories of traits for the Northeast Arctic stock of Atlantic cod for temperature scenarios 2 to 20°C. Phenotypic traits: length (a), gonad weight (b). Behavioural and life history strategies: foraging (c) and maturation age (d). Population-level consequences: natural mortality (e) and expected lifetime gonad production (f). Panels g-l are the same as a-f but use an ever-increasing function of maximum oxygen uptake (See, Fig. S2). Central line denotes the population mean value; shaded grey areas show within-population variance due to environmental stochasticity.

Foraging Survival Trade-Off

There are several reasons why increased foraging behaviour can result in increased mortality and decreased survival. The overarching of which is greater exposure to predators (Jørgensen & Holt, 2013; Holt & Jørgensen, 2014), however there are many other mechanisms or trade-offs that can also increase mortality and decrease survival when foraging is increased. Habitat choice for example can have an implication as habitats may differ in food availability and exposure to predators (Enberg *et al.*, 2012). The actual food items themselves can increase mortality and decrease survival. Food items differ in size, energy content, digestibility, presence of parasites. If foraging were increased, individuals may potentially ingest a higher volume of parasites, pathogens or toxins that may have long-term health and survival implications (Enberg *et al.*, 2012). Furthermore, if larger or smaller than the optimal size of prey are ingested, this may result in increased energy intake but also a cost of increased handling time during which individuals may be at higher risk of predation (Enberg *et al.*, 2012). During search time for prey, there may be a trade-off between ingesting an encountered food item or continuing to search for a more profitable one, all the while increasing exposure to predators (Enberg *et al.*, 2012). In terms of aerobic scope, digestion elevates metabolic rate occupying a larger proportion of aerobic scope that can be used for other purposes such as swimming. Increased foraging and digestion can therefore lead to reduced swimming performance and increased predation risk (Lankford *et al.*, 2001; Enberg *et al.*, 2012).

References

Claireaux G, Webber DM, Lagardére JP, Kerr SR 2000 Influence of water temperature and oxygenation on the aerobic metabolic scope of Atlantic cod (*Gadhus Morhua*). *J Sea Res.* **44**, 257–265. (doi: 10.1016/S1385-1101(00)00053-8)

Clarke A, Johnston NM 1999 Scaling of metabolic rate with body mass and temperature in teleost fish. *J Anim Ecol* 68, 893–905. (*doi*: 10.1046/j.1365-2656.1999.00337.x)

Enberg K, Jørgensen C, Dunlop ES, Varpe Ø, Boukal DS, Baulier L, Eliassen S, Heino M. 2012 Fishing-induced evolution of growth: concepts, mechanisms and the empirical evidence. *Mar Ecol.* **33**, 1-25. (doi:10.1111/j.1439-0485.2011.00460.x)

Holt RE, Jørgensen C.2014 Climate warming causes life-history evolution in a model for Atlantic cod (*Gadus morhua*). *Cons Phys.* **2**:cou050. (doi: 10.1093/conphys/cou050)

ICES (2012) Report of the Arctic Fisheries Working Group 2012 (AFWG), 20–26 April 2012, ICES Headquarters, Copenhagen. ICES CM 2012/ ACOM:05. 633 pp.

Jobling M (1988) A review of the physiological and nutritional energetics of cod, *Gadus morhua* L., with particular reference to growth under farmed conditions. *Aquaculture.* **70**:1–19. (doi: 10.1016/0044-8486(88)90002-6)

Jørgensen C, Holt RE. 2013 Natural mortality: Its ecology, how it shapes fish life histories and why it may be increased by fishing. *J Sea Res*. 75, 8-18 (doi:10.1016/j.seares.2012.04.003)

Lankford TE, Billerbeck JM, Conover DO. 2001 Evolution of intrinsic growth and energy acquisition rates II Trade-offs with vulnerability to predation in *Menidia menidia. Evolution.* **55**, 1873-1881. (doi/10.1111/j.0014-3820.2001.tb00836.x)
