# Supplementary material for: Climate change in fish: effects of respiratory constraints on optimal life history and behaviour
Source: Biol Lett. 2015 Feb;11(2):20141032. doi: 10.1098/rsbl.2014.1032 (PMC4360111; doi:10.1098/rsbl.2014.1032)
Supplement: Matlab Model Code, Climate change in Fish: Effects of respirometry constraints on optimal life-history and behaviour [file rsbl20141032supp2.docx]

**Supporting Information**

**Climate change in fish: effects of respiratory constraints on optimal life-history and behaviour**

Rebecca E. Holt and Christian Jørgensen

**Matlab Code**

close all;

clear all;

%Flags

RunClimateWarming = 0;

RunOptimization = 1;

%FIGURES to plot

PlotStrategies = 1;

PlotForward = 1;

% DEFINE PARAMETERS

%Resolution of model

LengthMax = 200; LengthStep = 2;

ForagingBehMin = 1; ForagingBehMax = 10; ForagingBehStep = 0.2;

AllocationStep = 0.05;

AgeMin = round(1); AgeMax = round(30);

EMax = 11;

IMax = 1000;

StepsWithinYear = 24;

K = 0.95; WeightMax = (K/1E5)*(LengthMax^3);

JKg= ((1104.538*4.1868)*1000); %J per Kg wet weight cod tissue (1cal=4.1868J)(Converting Cal.g wet weight to J.Kg wet weight). Data on cod body composition from Holdway and Beamish 1984, Table 2.

%Physiology

CostOfForaging = 0.15;

SDACoeff = 0.17; %J Kg y-1 Hansson et al 1996

%COT (Cost of Transport) - Migration

%Ware et al 1978 J KM

a = 4.18e+01;% J Yr-1

r = 0.138; %s-1

e = 0.43; %No Units - Scaling factor for optimal cruising swimming speed

b= 1.02; %No units - Scaling with length for cost of transport

ee =2.42; %Scaling with swim speed for COT

%Fisheries selectivity

UseSelectivity = 0;

Slope = 0.1; L50 = 30.;

%Natural Mortalities

SizeMVariable = 1;

SizeMCoeff = 0.33; SizeMExp = -0.75; %Size dependent mortality yr-1

SpawningMFactor_value = 0; %Extra mortality when spawning, yr-1

SizeIndependentM = 0.07; % Baseline mortality yr-1

O2M_coeff = 11; O2M_exp = 3; %Used Scope mortality yr-1

ForagingM_coeff = 0.030; ForagingM_exp = 3; %Foraging mortality

%Parameter Values - NEA Cod Parameters

LengthMin = 16;%Length when introduced to model at age 1 (cm)

Fishing = 0.17;%Annual mortality rate from fishing

DM = 780;% Spawning migration distance (km)

TMean = 4.0;%Mean experienced temperature (degC)

TPeak = 0.66;%Time of peak temperature (the year goes 0 to 1)

TAmp = 1.04;%Amplitude of annual temperature cycle (degC)

TOffsetMax = 3;%Maximum climate change offset to run

Productivity = 1;%Environmental Mean - Productivity

%Other physiological and ecological parameters

GSImax = 0.10; GonadExp = 2.5;

CostOfGonadTissue = 1.5;

ConversionEfficiency = 0.5;

if RunClimateWarming == 1;

ClimateChangeOffset = (0:1:TOffsetMax);

else

ClimateChangeOffset = 0;

end

%Temperature Seasonality

d = (0:1/StepsWithinYear:1);

Seasonality =(TMean+0.5*TAmp*cos((d-TPeak)*2*pi()));

[Seasonality,ClimateChangeOffset]= meshgrid(Seasonality,ClimateChangeOffset);

Temp = ClimateChangeOffset+Seasonality;

%Array dimensions

LMax = (LengthMax-LengthMin)/LengthStep+1; %Length

BehMax = (ForagingBehMax-ForagingBehMin)/ForagingBehStep+1; %Behaviour

AMax = 1/AllocationStep+1; %Allocation

% DECLARE VARIABLES

%Fitness and strategy matrices

F(1:EMax,1:LMax,AgeMin:AgeMax) = 0.;

Strategy(1:2,1:EMax,1:LMax,AgeMin:AgeMax) = 0.;

Fitness(1:AMax,1:BehMax) = 0.;

%Other variables

Age=round(0); L=round(0); B=round(0); A=round(0); Harvest=round(0); Step=round(0);

Length(1:EMax,1:AMax,1:BehMax)=0.; intL(1:EMax,1:AMax,1:BehMax)=round(0); dL(1:EMax,1:AMax,1:BehMax)=0.; NewL(1:EMax,1:AMax,1:BehMax)=0.;

Weight(1:EMax,1:AMax,1:BehMax)=0.; Gain(1:EMax,1:AMax,1:BehMax)=0.; Gonads(1:EMax,1:AMax,1:BehMax)=0.; GSI = 0.;

SizeM(1:EMax,1:AMax,1:BehMax)=0.; SizeM_step(1:EMax,1:AMax,1:BehMax)=0.;

O2M(1:EMax,1:AMax,1:BehMax)=0.; O2M_step(1:EMax,1:AMax,1:BehMax)=0.; UsedScope(1:EMax,1:AMax,1:BehMax)=0.;

GonadM(1:EMax,1:AMax,1:BehMax)=0.; ForagingM(1:EMax,1:AMax,1:BehMax)=0.; SpawningM(1:EMax,1:AMax,1:BehMax)=0.; SpawningMFactor(1:EMax,1:AMax,1:BehMax)=0.;

FishingM(1:EMax,1:AMax,1:BehMax)=0.; Selectivity(1:EMax,1:AMax,1:BehMax) = 0.;

Survival(1:EMax,1:AMax,1:BehMax)=0.; Intake(1:EMax,1:AMax,1:BehMax)=0.; NetIntake(1:EMax,1:AMax,1:BehMax)=0.;

SDA(1:EMax,1:AMax,1:BehMax)=0.; EnergeticCostOfForaging(1:EMax,1:AMax,1:BehMax)=0.;

Strat1(1:LMax,1:AgeMax-1) = 0.; Strat2(1:LMax,1:AgeMax-1) = 0.;

EnergeticForagingRateCoeff(1:EMax,1:AMax,1:BehMax) = 0.; EnergeticForagingCostCoeff(1:EMax,1:AMax,1:BehMax) = 0.;

%Forward simulation

Ind(1:16,AgeMin:AgeMax,1:IMax,1:size(Temp,1)) = 0.;

MeanInd(1:19,AgeMin:AgeMax,1:size(Temp,1)) =0.;

iLength = 0.; iintL = round(0); idL = 0.;

iWeight = 0.; iGonads = 0.; iGSI = 0.;

iAllocation = 0.; iForagingBehaviour = 0.; iForagingRate = 0.; iIntake = 0.; iROSM = 0.;

iSizeM = 0.; iSizeM_step = 0.; iForagingM = 0.; iGonadM = 0.; iSpawningM = 0.; iSurvival = 0.;

iFishingM = 0.; iSelectivity = 0.; iRespiration = 0.; iNetIntake=0.;

%FIGURE handles etc

hStrat = 1; hStrat1 = 0.; hStrat1surf = 0.; hStrat2 = 0.; hStrat2surf = 0.;

hForward = 2;

hFigure2 = 3;

hFigure1a = 3;

hFigure1c = 3;

hFigure1b = 3;

hSeasonality = 3;

%Calculating foraging risk and allocation matrices for array-based

%optimization

clear('ForagingBehaviour');

ForagingBehaviour(1:AMax,1:BehMax) = 0.;

for A = 1:AMax;

ForagingBehaviour(A,1:BehMax)=ForagingBehMin:ForagingBehStep:ForagingBehMax;

end

Allocation(1:AMax,1:BehMax) = 0.;

for B = 1:BehMax

Allocation(1:AMax,B)=0:AllocationStep:1;

end

%Respiration Physiology

% Clarke & Johnston 1999 - General teleost fish

SMR_exp_CJ = 0.80;

SMR_coeff_CJ = exp(-5.43); %mmol O2 g-1 h-1

SMR_coeff_CJ = SMR_coeff_CJ * 434 * 24 *365; %J g-1 y-1

SMR_coeff_CJ = SMR_coeff_CJ * (0.001^(-SMR_exp_CJ)); %J kg-1 y-1

%Correction acording to new exponent

SMR_exp = 0.70;

StandardWeight = 3; %kg

SMR_coeff_changedexp = SMR_coeff_CJ * (StandardWeight^(SMR_exp_CJ-SMR_exp));

%Temperature effect on SMR

SMR_std = SMR_coeff_changedexp*(0.05^SMR_exp); %50g fish

SMR_TempFunction = exp(15.7-(5.02.*1000./(Temp+273.15))).* 434 .* 24 .*365;

SMR_TempFunction = SMR_TempFunction./SMR_std;

SMR_coeff=SMR_coeff_changedexp*SMR_TempFunction;

%Adapted Claireaux et al. 2000

AMR_TempFunction = (3.15*10^7 - 2.9*10^7.*exp(-0.12.*Temp) - 660*exp(0.5*Temp));

% AMR_TempFunction = (3.15*10^7 - 2.9*10^7.*exp(-0.12.*Temp) - 600*exp(0.1*Temp)); %Sensitivity Analysis

AMR_exp = SMR_exp;

AMR_coeff = AMR_TempFunction;

%Temperature effect on foraging Input

StdTemp = 4; Magnitude = 0;

SMR_TempFunctionAtStdTemp = exp(15.7-(5.02.*1000./(StdTemp+273.15))) .* 434 .* 24 .*365/SMR_std;

SMR_AtStdTemp = SMR_coeff_changedexp * SMR_TempFunctionAtStdTemp; %J kg-SMR_exp y-1

Foraging_TempFunction = ((1-Magnitude) + Magnitude.*(SMR_TempFunction./SMR_TempFunctionAtStdTemp)).*SMR_AtStdTemp;

%Environmental Stochasticity

EStDev = 2.5;

EMean = 1+(EMax-1)/2;

ProbE(1:11) = 0.; ValueE(1:11) = 0.;

ScaleE = 0.3;

for E = 1:EMax;

ProbE(E) = (1/(EStDev*sqrt(2*pi())))*exp(-((E-EMean)^2)/(2*(EStDev^2)));

ValueE(E) = 1+ScaleE*(2*((E-1)/(EMax-1))-1);

end

sumProbE = sum(ProbE);

ProbE(:) = ProbE(:)/sumProbE;

for E = 1:EMax;

EnergeticForagingRateCoeff(E,:,:) = ValueE(E) .* Productivity .* ForagingBehaviour(:,:);

EnergeticForagingCostCoeff(E,:,:) = ForagingBehaviour(:,:) .* CostOfForaging ;

end

clear ('ForagingBehaviour', 'Allocation');

ForagingBehaviour(1:EMax,1:AMax,1:BehMax) = 0.;

Allocation(1:EMax,1:AMax,1:BehMax) = 0.;

for E = 1:EMax;

for A = 1:AMax;

ForagingBehaviour(E,A,1:BehMax)=ForagingBehMin:ForagingBehStep:ForagingBehMax;

end

for B = 1:BehMax

Allocation(E,1:AMax,B)=0:AllocationStep:1;

end

end

%Backwards Iteration and Optimization

for TemperatureNo = 1:size(Temp,1);

if (RunClimateWarming == 1) || (RunOptimization == 1);

F(:,:,:) = 0.;

Strategy(:,:,:,:) = 0.;

SpawningMFactor(:,:,:) = SpawningMFactor_value;

SpawningMFactor(:,1,:) = 0.;

if PlotStrategies == 1;

hStrat1 = subplot(1,2,1);

[X,Y] = meshgrid(AgeMin:AgeMax-1,LengthMin:LengthStep:LengthMax);

hStrat1surf = surf(X,Y,Strat1);

set(hStrat1surf,'ZDataSource','Strat1');

xlabel('Age (years)');

ylabel('Length (cm)');

title('Allocation');

axis([AgeMin AgeMax-1 LengthMin LengthMax 0 1]); axis square; caxis([0 1]);

hStrat2 = subplot(1,2,2);

hStrat2surf = surf(X,Y,Strat2);

set(hStrat2surf,'ZDataSource','Strat2');

xlabel('Age (years)');

ylabel('Length (cm)');

title('Foraging risk');

axis([AgeMin AgeMax-1 LengthMin LengthMax 0 10]); axis square; caxis([0 2]);

end % PlotStrategies

for Age = AgeMax-1:-1:AgeMin

for L = 1:LMax;

Length(:,:,:) = LengthMin + (L-1)*LengthStep; %cm

Weight(:,:,:) = (K/1E5).*(Length(:,:,:).^3);

Gonads(:,:,:) = 0.;

SizeM(:,:,:) = 0.;

FishingM(:,:,:)=0.;

GonadM(:,:,:) = 0.;

O2M(:,:,:) = 0.;

for Step=1:StepsWithinYear;

SizeM_step(:,:,:) = (1./StepsWithinYear).*SizeMCoeff .* (Length(:,:,:).^SizeMExp);

SizeM(:,:,:) = SizeM(:,:,:) + SizeM_step(:,:,:);

if UseSelectivity == 1

Selectivity(:,:,:) = 1./(1+exp(-Slope.*(Length(:,:,:)-L50)));

else

Selectivity(:,:,:) = 1.;

end

FishingM(:,:,:) = FishingM(:,:,:) + (1./StepsWithinYear).*Fishing.*Selectivity(:,:,:); %Fishing mortality y-1

EnergeticCostOfForaging(:,:,:) = EnergeticForagingCostCoeff(:,:,:).* SMR_coeff(1, Step).*(Weight(:,:,:).^SMR_exp); %J y-1.

Intake(:,:,:) = EnergeticForagingRateCoeff(:,:,:) .* Foraging_TempFunction(TemperatureNo, Step) .* (Weight(:,:,:).^SMR_exp); % J y-1.

SDA(:,:,:) = SDACoeff.*Intake(:,:,:); %J y-1

SMR(:,:,:) = SMR_coeff(TemperatureNo, Step).*((Weight(:,:,:)+Gonads(:,:,:)).^SMR_exp); %J y-1

SMR_somatic(:,:,:) = SMR_coeff(TemperatureNo, Step).*((Weight).^SMR_exp); %J y-1

SMR_gonadal(:,:,:) = SMR_coeff(TemperatureNo, Step).*((Gonads.^SMR_exp)); %J y-1

AMR(:,:,:) = AMR_coeff(TemperatureNo, Step).*(Weight(:,:,:).^AMR_exp); %J y-1

NetIntake(:,:,:) = (Intake(:,:,:) - SMR(:,:,:) - EnergeticCostOfForaging(:,:,:) - SDA(:,:,:)); %J y-1

NetIntake_somatic(:,:,:) =(Intake(:,:,:) - SMR_somatic(:,:,:) - EnergeticCostOfForaging(:,:,:) - SDA(:,:,:)); %J y-1

NetIntake_gonadal(:,:,:) =(Intake(:,:,:) - SMR_gonadal(:,:,:) - EnergeticCostOfForaging(:,:,:) - SDA(:,:,:)); %J y-1

EnergeticCostOfGrowth(:,:,:) = (1-ConversionEfficiency).*NetIntake(:,:,:); %J y-1

EnergeticCostOfGrowth_somatic(:,:,:) = (1-ConversionEfficiency).*NetIntake_somatic(:,:,:); %J y-1

EnergeticCostOfGrowth_gonadal(:,:,:) = (1-ConversionEfficiency).*NetIntake_gonadal(:,:,:); %J y-1

NetIntake(:,:,:) = NetIntake(:,:,:) - EnergeticCostOfGrowth(:,:,:); %J y-1

PositiveIntake(:,:,:) = (NetIntake(:,:,:) > 0);

%Simple Oxygen Budget

UsedScope(:,:,:) = SMR(:,:,:) + EnergeticCostOfForaging(:,:,:) + SDA(:,:,:) + EnergeticCostOfGrowth(:,:,:); %J y-1

O2M_step(:,:,:) = O2M_coeff.*(UsedScope(:,:,:)./AMR(:,:,:)).^O2M_exp;

O2M(:,:,:) = O2M(:,:,:) + O2M_step(:,:,:).*SizeM_step(:,:,:);

%Allocation and growth

Weight(:,:,:) = Weight(:,:,:) + ...

PositiveIntake(:,:,:) .*(1./StepsWithinYear).*(1-Allocation(:,:,:)).*NetIntake(:,:,:)./JKg + ...

(1-PositiveIntake(:,:,:)).*(1./StepsWithinYear).*NetIntake(:,:,:)./JKg; %kg step-1

Length(:,:,:) = min((1E5.*Weight(:,:,:)./K).^(1/3), LengthMax);

Weight(:,:,:) = min(Weight(:,:,:), WeightMax);

Gonads(:,:,:) = Gonads(:,:,:) + PositiveIntake(:,:,:).*(1./StepsWithinYear).*max(0, (1./CostOfGonadTissue).*Allocation(:,:,:).*NetIntake(:,:,:)./JKg); %kg step-1

GonadM(:,:,:) = GonadM(:,:,:) + SizeM_step(:,:,:).*(((Gonads(:,:,:)./(Weight(:,:,:)+Gonads(:,:,:)))./GSImax).^GonadExp);

end %Steps within year

%Energetic Cost of Migration

UOpt(:,:,:)= (r.*(Length(:,:,:).^e)); %Optimal swimming speed dependent on length of fish taken from Ware 1978

COTware(:,:,:) =(a.*(Length(:,:,:).^b).*(UOpt(:,:,:).^ee)); % J/Km - using length at the end of the year (Energetic Cost of Transport)

Migration(:,:,:) = (1./CostOfGonadTissue).*(COTware(:,:,:).*(2*DM))/JKg;% was J - divided by Jkg to convert to kg GONADS step-1 - Migration occurs at the end of the year

Migration(:,1,:) = 0.;

Gonads(:,:,:) = max(0., (Gonads(:,:,:) - Migration(:,:,:)));

ForagingM(:,:,:) = ForagingM_coeff.*(ForagingBehaviour(:,:,:).^ForagingM_exp).*SizeM(:,:,:);

SpawningM(:,:,:) = SpawningMFactor(:,:,:).*(SizeMCoeff .* (Length(:,:,:).^SizeMExp));

Survival(:,:,:) = exp(-ForagingM(:,:,:) - FishingM(:,:,:) - SizeIndependentM - SizeM(:,:,:) - GonadM(:,:,:) - O2M(:,:,:) - SpawningM(:,:,:));

intL(:,:,:) = max(1, min(floor((Length(:,:,:)-LengthMin)./LengthStep)+1, LMax-1));

dL(:,:,:) = ((Length(:,:,:)-LengthMin)./LengthStep)+1-intL;

for E = 1:EMax

Fitness(:,:) = 0.;

for A = 1:AMax;

for B = 1:BehMax;

for EnextT = 1:EMax

Fitness(A,B) = Fitness(A,B) + ProbE(EnextT)*(dL(E,A,B)*F(EnextT,intL(E,A,B)+1,Age+1) + (1-dL(E,A,B))*F(EnextT,intL(E,A,B),Age+1)); %Residual reproductive value = future fitness

end

end

end

Fitness(:,:) = squeeze(Survival(E,:,:)) .* (Fitness(:,:) + squeeze(Gonads(E,:,:))); %Reproduction at the end of year, gonads added to residual fitness, everything discounted by survival

[OptAFitness,OptA] = max(Fitness); %First find optimal A (for each B)- matlab stores optimal fitness and index of optimal fitness in the two arrays

[OptFitness,OptB] = max(OptAFitness,[],2); %Then find optimal B as maximum of the many values from previous line.

F(E,L,Age) = OptFitness; %Optimal fitness (States age and lenght as well as the environment)

Strategy(1,E,L,Age) = (OptA(OptB)-1)*AllocationStep; %Allocation strategy

Strategy(2,E,L,Age) = ForagingBehMin+(OptB-1)*ForagingBehStep; %Foraging strategy

end %E

end %Length

if PlotStrategies == 1;

Strat1 = reshape(Strategy(1,6,:,AgeMin:AgeMax-1),LMax,[]);

Strat2 = reshape(Strategy(2,6,:,AgeMin:AgeMax-1),LMax,[]);

refreshdata(hStrat1surf);

refreshdata(hStrat2surf);

drawnow;

end

end %Age

end %Run optimization

% FORWARD SIMULATION - Allows visualization of the individual and population level characteristics emerging from the interaction of the life history strategy and the environment

%Initiate first cohort

iLength = LengthMin; %

iWeight = (K/1E5)*(iLength^3);

iSurvival = 1.;

Ind( 1,AgeMin,:,TemperatureNo) = iLength; % 1: Length at age %Ind specifies the trait (1(length,for all age min to max,for all temps)

Ind( 2,AgeMin,:,TemperatureNo) = iWeight; % 2. Weight at age

Ind( 3,AgeMin,:,TemperatureNo) = 1.; % 3. Survival until age

% Ind( 4,AgeMin,I,TemperatureNo) = 0.; % 4. Gonads at end of age

% Ind( 5,AgeMin,I,TemperatureNo) = 0.; % 5. GSI

% Ind( 6,AgeMin,I,TemperatureNo) = 0.; % 6. Allcoation value

% Ind( 7,AgeMin,I,TemperatureNo) = 0.; % 7. Risk taken

% Ind( 8,AgeMin,I,TemperatureNo) = 0.; % 8. Intake

% Ind( 9,AgeMin,I,TemperatureNo) = 0.; % 9. Environmental value

% Ind(10,AgeMin,I,TemperatureNo) = 0.; %10. Size-dependent predation mortality

% Ind(11,AgeMin,I,TemperatureNo) = 0.; %11. Fishing mortality

% Ind(12,AgeMin,I,TemperatureNo) = 0.; %12. Foraging mortality

% Ind(13,AgeMin,I,TemperatureNo) = 0.; %13. Gonad mortality

% Ind(14,AgeMin,I,TemperatureNo) = 0.; %14. ROSM

% Ind(15,AgeMin,I,TemperatureNo) = 0.; %15. Total Natural Mortality

% Ind(16,AgeMin,I,TemperatureNo) = 0.; %16. Oxygen mortality

% Ind(17,AgeMin,I,TemperatureNo) = 0.; %17. SMR

% Ind(18,AgeMin,I,TemperatureNo) = 0.; %18. AMR

% Ind(19,AgeMin,I,TemperatureNo) = 0.; %19. SpawningM

%

for I = 1:IMax;

iLength = LengthMin;

iWeight = (K/1E5)*(iLength^3);

iSurvival = 1.;

for Age = AgeMin:AgeMax-1;

EisOK = 0.;

while EisOK==0;

iE = EMean + EStDev*randn(1);

if iE > 1;

if iE < EMax;

EisOK = 1;

end

end

end

EValue = 1+ScaleE*(2*((iE-1)/(EMax-1))-1);

%Look up optimal strategy

iintE = max(0.,min(floor(iE),EMax-1));

idE = max(0.,min(iE-iintE,1));

iintL = max(1, min(floor((iLength-LengthMin)./LengthStep)+1, LMax-1));

idL = (iLength-LengthMin)./LengthStep+1-iintL;

iAllocation = idE *idL*Strategy(1,iintE+1,iintL+1,Age) + idE *(1.-idL)*Strategy(1,iintE+1,iintL ,Age) + ...

(1.-idE)*idL*Strategy(1,iintE ,iintL+1,Age) + (1.-idE)*(1.-idL)*Strategy(1,iintE ,iintL ,Age);

iAllocation = max(0., min(iAllocation, 1.));

iForagingBehaviour = idE *idL*Strategy(2,iintE+1,iintL+1,Age) + idE *(1.-idL)*Strategy(2,iintE+1,iintL ,Age) + ...

(1.-idE)*idL*Strategy(2,iintE ,iintL+1,Age) + (1.-idE)*(1.-idL)*Strategy(2,iintE ,iintL ,Age);

iSizeM = 0.;

iFishingM=0.;

iGonads = 0.;

iGonadM = 0.;

iO2M = 0.;

iEnergeticForagingRateCoeff = iForagingBehaviour*EValue*Productivity;

iEnergeticForagingCostCoeff = iForagingBehaviour*CostOfForaging; % Same unit as SMR_coeff: J kg-SMR_exp y-1

for Step=1:StepsWithinYear;

iSizeM_step = (1/StepsWithinYear)*SizeMCoeff * (iLength^SizeMExp);

iSizeM = iSizeM + iSizeM_step;

if UseSelectivity == 1;

iSelectivity = 1/(1+exp(-Slope*(iLength-L50)));

else

iSelectivity = 1;

end

iFishingM = iFishingM + (1/StepsWithinYear)*Fishing*iSelectivity; %Fisheries mortality %J y-1

iEnergeticCostOfForaging = iEnergeticForagingCostCoeff*SMR_coeff(1, Step)*(iWeight^SMR_exp); %J y-1

iIntake = iEnergeticForagingRateCoeff*Foraging_TempFunction(TemperatureNo, Step)*(iWeight^SMR_exp); %J y-1

iSDA = SDACoeff*iIntake; %J y-1

iSMR = SMR_coeff(TemperatureNo, Step)*((iWeight+iGonads)^SMR_exp); %J y-1

iSMR_somatic = SMR_coeff(TemperatureNo, Step)*((iWeight)^SMR_exp); %J y-1

iSMR_gonadal = SMR_coeff(TemperatureNo, Step)*((iGonads^SMR_exp)); %J y-1

iAMR = AMR_coeff(TemperatureNo, Step)*(iWeight^AMR_exp); %J y-1

iNetIntake = iIntake - iSMR - iEnergeticCostOfForaging - iSDA; %J y-1

iNetIntake_somatic =(iIntake - iSMR_somatic - iEnergeticCostOfForaging - iSDA); %J y-1

iNetIntake_gonadal =(iIntake - iSMR_gonadal- iEnergeticCostOfForaging- iSDA); %J y-1

iEnergeticCostOfGrowth = (1-ConversionEfficiency)*iNetIntake; %J y-1 taking into account assimilation efficiency

iEnergeticCostOfGrowth_somatic = (1-ConversionEfficiency)*iNetIntake_somatic; %J y-1

iEnergeticCostOfGrowth_gonadal = (1-ConversionEfficiency)*iNetIntake_gonadal; %J y-1

iNetIntake = iNetIntake - iEnergeticCostOfGrowth; %J y-1

iPositiveIntake = (iNetIntake) > 0; %J y-1

iUsedScope = iSMR + iEnergeticCostOfForaging + iSDA + iEnergeticCostOfGrowth; %J y-1

iO2M_step = O2M_coeff*((iUsedScope/iAMR)^O2M_exp); %Used scope respiration cost yr-1

iO2M = iO2M + iO2M_step*iSizeM_step; %yr-1

if iNetIntake >0

iSomaticGrowth = (1/StepsWithinYear)*(1-iAllocation)*iNetIntake/JKg; %kg step-1

iGonadGrowth = (1/StepsWithinYear)*max(0, (1/CostOfGonadTissue)*iAllocation*iNetIntake/JKg); %kg step-1

else

iSomaticGrowth = (1/StepsWithinYear)*iNetIntake/JKg; %kg step-1

iGonadGrowth = 0.; %kg step-1

end

iWeight = max(0., min(iWeight+ iSomaticGrowth,WeightMax)); %kg

iLength = min((1E5*iWeight/K)^(1/3), LengthMax); %cm

iWeight = min(iWeight,WeightMax);%kg

iGonads = iGonads + iGonadGrowth;%kg

iGonadM = iGonadM + iSizeM_step*(((iGonads/(iWeight+iGonads))/GSImax)^GonadExp); %yr-1

end %Steps within year

%Energetic Cost of Migration

iUOpt= r*(iLength^e); %Size dependent

iCOTware =(a*(iLength^b)*(iUOpt^ee)); % J/m

iMigration = 0;

iSpawningM = 0;

if iAllocation > 1.e-10;

iMigration = (1/CostOfGonadTissue)*(iCOTware*(2*DM))/JKg;%kg gonads step-1

iSpawningM = SpawningMFactor_value*(SizeMCoeff * (iLength^SizeMExp));

end

iGonads = max(0,(iGonads - iMigration));

iForagingM = ForagingM_coeff*(iForagingBehaviour^ForagingM_exp)*iSizeM;

iSurvival = iSurvival * exp(-iForagingM - iFishingM - SizeIndependentM - iSizeM - iGonadM - iO2M - iSpawningM);

iGSI = (iGonads / (iWeight + iGonads));

Ind( 1,Age+1,I,TemperatureNo) = iLength;

Ind( 2,Age+1,I,TemperatureNo) = iWeight+iGonads;

Ind( 3,Age+1,I,TemperatureNo) = iSurvival;

Ind( 4,Age+1,I,TemperatureNo) = iGonads;

Ind( 5,Age+1,I,TemperatureNo) = iGSI;

Ind( 6,Age ,I,TemperatureNo) = iAllocation;

Ind( 7,Age ,I,TemperatureNo) = iForagingBehaviour;

Ind( 8,Age ,I,TemperatureNo) = iForagingRate;

Ind( 9,Age ,I,TemperatureNo) = iE;

Ind(10,Age ,I,TemperatureNo) = SizeMVariable*iSizeM;

Ind(11,Age ,I,TemperatureNo) = iFishingM;

Ind(12,Age ,I,TemperatureNo) = iForagingM;

Ind(13,Age ,I,TemperatureNo) = iGonadM;

Ind(14,Age ,I,TemperatureNo) = iUsedScope/iAMR;

Ind(15,Age ,I,TemperatureNo) = iForagingM+SizeIndependentM+iGonadM+iO2M+iSpawningM+iSizeM;

Ind(16,Age ,I,TemperatureNo) = iO2M;

Ind(17,Age ,I,TemperatureNo) = iSMR;

Ind(18,Age ,I,TemperatureNo) = iAMR;

Ind(19,Age ,I,TemperatureNo) = iSpawningM;

Ind(20,Age ,I,TemperatureNo) = iSDA;

Ind(21,Age ,I,TemperatureNo) = iEnergeticCostOfForaging;

Ind(22,Age ,I,TemperatureNo) = iSMR_somatic;

Ind(23,Age ,I,TemperatureNo) = iSMR_gonadal;

Ind(24,Age ,I,TemperatureNo) = iEnergeticCostOfGrowth_somatic;

Ind(25,Age ,I,TemperatureNo) = iEnergeticCostOfGrowth_gonadal;

Ind(26,Age ,I,TemperatureNo) = iEnergeticCostOfGrowth;

end %Age

end % Individual

for Trait = 1:26;

for Age = AgeMin:AgeMax;

MeanInd(Trait,Age,TemperatureNo) = sum(Ind(Trait,Age,:,TemperatureNo))/IMax;

StDevInd(Trait,Age,TemperatureNo) = sqrt(sum((Ind(Trait,Age,:,TemperatureNo)-MeanInd(Trait,Age,TemperatureNo)).^2)/(IMax-1));

StDevPlus(Trait,Age,TemperatureNo) = (sum(Ind(Trait,Age,:,TemperatureNo))/IMax) + (sqrt(sum((Ind(Trait,Age,:,TemperatureNo)-Ind(Trait,Age,TemperatureNo)).^2)/(IMax-1)));

StDevminus(Trait,Age,TemperatureNo) = (sum(Ind(Trait,Age,:,TemperatureNo))/IMax) - (sqrt(sum((Ind(Trait,Age,:,TemperatureNo)-Ind(Trait,Age,TemperatureNo)).^2)/(IMax-1)));

end

end

end %Temperature
